# The Engineer's Department at Guy's Hospital

**Published:** 1916-01-15

**Authors:** 


### HOSPITAL REPAIRS AND MAINTENANCE.

### The Engineer's Department at Guy's Hospital.

The following report of the consulting engineer to Guy's Hospital (Mr. Thomas Kirkland) on the accounts for its central station for the year 1915 is most suggestive and full of interesting details. He announces the completion within the period of the orthopædic and tuberculous out-patient departments and of a new bacteriological department. Further, that the kitchen for the Henriette Raphael Nursing Home has been remodelled and enlarged, so as to include separate rooms for washing-up, for preparing vegetables and for cooking, together with three cold-storage rooms. The latter facts recall to mind the thorough organisation and attention to detail which are essential to success in the equipment of the nursing establishment of a great hospital in the present day.

### Engine-house Plant.

Additional refrigerating plant being required to deal with the cold-storage rooms in the Nurses' Home and with the cold-storage room in the bacteriological department, it was decided to remove the plant which had hitherto been used for the mortuary and pathological department, as this was too small to do the extra work required. The new plant, consisting of two motor-driven ammonia compressors, condensers, evaporator, brine-pipes, tank, etc., has been placed in the extension of the engine-room under Tabard House, where it is under the direct control of the engineering staff. This has now been completed and furnishes the means for cooling the mortuary chambers, the cold rooms in the pathological department, the bacteriological department, and the Nurses' Home kitchen, and provision has been left for extensions as may be required.

The 120 k.w. set which I stated last year had been re-erected has been set to work and found very useful in connection with the night load of the hospital. The main generating plant has been kept in good repair.

### Boiler-house Cost and Coal Consumption.

During the year one of the economisers has been renewed. The boilers and the rest of the plant have been kept in good condition and have been tested and passed as satisfactory by the Vulcan Boiler Insurance Company. The amount of coal used in the central station in each of the last six years is shown by the following statement:—

| Year | Amount in Tons | Cost | Remarks |
|---|---|---|---|
| 1910 | ... 4,437 | £3,665 7 10 | |
| 1911 | ... 4,456 | £3,694 4 2 | |
| 1912 | ... 4,641 | £4,217 3 9 | Coal and transport workers' strikes. |
| 1913 | ... 4,984 | £4,993 14 3 | Coal dearer as result of strikes. |
| 1914 | ... 5,114 | £5,206 8 5 | War declared on August 4. |
| 1915 | ... 5,445 | £6,966 19 5 | War. |

It will be seen that more coal has been used during the year, amounting to 331 tons, which is caused by increased demands in the hospital. The cost of the coal has increased by £1,760 11s. This large additional cost is mainly due to the increase in price since the war.

### Electric Light and Power.

*Summary of Electricity used and Cost per Annum.*

| Year | Units of Electricity used in the Hospital | Cost per Unit (exclusive of Interest and Depreciation) |
|---|---|---|
| 1911 ... ... | 302,450 | 0.98484d. |
| 1912 ... ... | 313,710 | 1.01507d. |
| 1913 ... ... | 327,030 | 1.00526d. |
| 1914 ... ... | 336,889 | 1.07682d. |
| 1915 ... ... | 337,650 | 1.11026d. |

Two hundred and fifty additional lights were connected during the year, bringing the total number to about 5,330 in use in the hospital. Ninety-six additional plugs were connected, making a total of about 1,336 plugs. Eight additional motors have been installed, which include five in connection with the refrigerating plant, together with one driving the plate-washer in the kitchen, one driving a centrifuge in the bacteriological department, and one in the electricians' shop. The motor driving the former refrigerating plant has been removed. More than six miles of wiring have been used in connection with these extensions, of which about one-half has been lead covered. The extra amount of current used during the year is 761 Board of Trade units. The cost per unit has increased from 1.07682d. to 1.11026d. This increase is due to the extra cost of coal. Maintenance charges are considerably less than last year, as fewer repairs and renewals have been required.

The smaller number of lamps used and the reduced cost is satisfactory and testifies to the supervision which the clerk of works has exercised in dealing with these stores.

*Summary of Electric Lamps issued from Stock and Cost per Annum.*

| Year | Metallic Filament | Cost | Carbon | Cost | Total Number | Total Cost per Annum |
|---|---|---|---|---|---|---|
| 1913 | 1,980 | £181 10 0 | 1,850 | £46 5 0 | 3,830 | £227 15 0 |
| 1914 | 1,792 | £155 13 8 | 1,761 | £44 0 6 | 3,553 | £199 14 2 |
| 1915 | 1,639 | £105 18 3 | 1,031 | £25 15 6 | 2,670 | £130 13 9 |

### Heating and Hot-water Supplies.

The heating and hot-water supplies generally have proved satisfactory. The additions made during the year involved the use of about 2½ miles of new piping.

### Bakery.

The machinery in the bakery has worked satisfactorily, and it may be of interest to note that 51,116 quartern loaves of bread have been made and baked during the year, in addition to 11,756 buns and 152 lb. of cake.

### Surgical Cutler's Shop.

This has proved increasingly useful, the number of instruments dealt with during the year amounting to 9,622, as against 8,302 for the previous year.

### Electro-plating.

The arrangements for electro-plating, which were nearly completed during the previous year, have now had some twelve months of work, and have been of considerable use, as will be seen from the following list of articles which have been dealt with:—1,050 surgeons' instruments, 310 hot and cold water taps, 7 infusion stands, 13 sterilisers and boracic pots, pipes and fittings, 6 metal operating stools, 4 forceps pots and brackets, 6 rings for bowls, 4 sets foot-rest fittings for couches, 1 instrument tray and stand, 12 towel rollers and fittings, 100 feet of copper pipe, 12 test-tube racks, 12 hypodermic stands, 4 large surgical leg-splints, 20 plug washers and chains, 12 stays and chains, 72 anti-splashers for taps, 6 sets electric lamps and fittings. In addition to the convenience of getting this work carried out on the premises as and when required, the cost is considerably less than the hospital would have had to pay had these articles been sent away. The actual cost incurred for labour and materials for plating the above was about £74.

Laundry.

The machinery in the laundry has been kept in good repair. The calender beds have been tested and passed as satisfactory by the Vulcan Boiler Insurance Company.

